# MicroRNAs and Extracellular Vesicles as Distinctive Biomarkers of Precocious and Advanced Stages of Breast Cancer Brain Metastases Development

**DOI:** 10.3390/ijms22105214

**Published:** 2021-05-14

**Authors:** Inês Figueira, Joana Godinho-Pereira, Sofia Galego, Joana Maia, János Haskó, Kinga Molnár, Rui Malhó, Bruno Costa-Silva, Imola Wilhelm, István A. Krizbai, Maria Alexandra Brito

**Affiliations:** 1 Research Institute for Medicines (iMed.ULisboa), Faculty of Pharmacy, Universidade de Lisboa, 1649-003 Lisbon, Portugal; ifigueira@farm-id.pt (I.F.); joanagpereira@ff.ulisboa.pt (J.G.-P.); sofiagalego11@hotmail.com (S.G.); 2 Farm-ID—Associação da Faculdade de Farmácia para a Investigação e Desenvolvimento, 1649-003 Lisbon, Portugal; 3 Faculty of Pharmacy, Universidade de Lisboa, 1649-003 Lisbon, Portugal; 4 Champalimaud Centre for the Unknown, Champalimaud Foundation, 1400-038 Lisbon, Portugal; joana.maia@research.fchampalimaud.org (J.M.); bruno.costa-silva@research.fchampalimaud.org (B.C.-S.); 5 Graduate Program in Areas of Basic and Applied Biology, University of Porto, 4099-002 Porto, Portugal; 6 Biological Research Centre, Eötvös Loránd Research Network (ELKH), Institute of Biophysics, 6726 Szeged, Hungary; hasko.janos@brc.hu (J.H.); molnar.kinga@brc.hu (K.M.); wilhelm.imola@brc.hu (I.W.); krizbai.istvan@brc.hu (I.A.K.); 7 BioISI, BioSystems and Integrative Sciences Institute, Faculty of Sciences, Universidade de Lisboa, 1749-016 Lisbon, Portugal; rmmalho@fc.ul.pt; 8 Institute of Life Sciences, Vasile Goldis Western University of Arad, 310025 Arad, Romania

**Keywords:** tumor biomarker, blood-brain barrier, extracellular vesicles, miR-194-5p, miR-205-5p, breast cancer brain metastasis, triple negative breast cancer

## Abstract

Triple negative breast cancer presents higher mortality and poorer survival rates than other breast cancer (BC) types, due to the proneness to brain metastases formation, which are usually diagnosed at advanced stages. Therefore, the discovery of BC brain metastases (BCBM) biomarkers appears pivotal for a timely intervention. With this work, we aimed to disclose microRNAs (miRNAs) and extracellular vesicles (EVs) in the circulation as biomarkers of BCBM formation. Using a BCBM animal model, we analyzed EVs in plasma by nanoparticle tracking analysis and ascertained their blood-brain barrier (BBB) origin by flow cytometry. We further evaluated circulating miRNAs by RT-qPCR and their brain expression by in situ hybridization. In parallel, a cellular model of BCBM formation, combining triple negative BC cells and BBB endothelial cells, was used to differentiate the origin of biomarkers. Established metastases were associated with an increased content of circulating EVs, particularly of BBB origin. Interestingly, deregulated miRNAs in the circulation were observed prior to BCBM detection, and their brain origin was suggested by matching alterations in brain parenchyma. In vitro studies indicated that miR-194-5p and miR-205-5p are expressed and released by BC cells, endothelial cells and during their interaction. These results highlight miRNAs and EVs as biomarkers of BCBM in early and advanced stages, respectively.

## 1. Introduction

In 2020, more than two million new cases of breast cancer (BC) were diagnosed worldwide, comprising the most frequent malignancy among women and being responsible for 684,996 deaths [1]. Metastatic brain tumors from BC affect approximately 15% of patients [2], with a propensity of circa 50% in triple negative BC (TNBC) [3] and a poor prognosis, with reports of 1-year survival of only 20% [4]. For this dramatic scenario contributes the lack of reliable peripheral biomarkers of BC brain metastases (BCBM) during disease progression, culminating with the disease only being diagnosed at advanced stages. Undeniably, there is an urgent unmet need for the discovery of biomarkers of BCBM formation.

Ideally, a highly predictive biomarker must be reproducible, sensitive, stable, specific, and easy to detect in tissues and/or body fluids [5]. Extracellular vesicles (EVs) and/or small, non-coding regulatory micro RNAs (miRNAs or miR) found in the circulation encompass new candidate disease biomarkers worth exploring. In fact, both can be detected in body fluids, holding a role as potential tumor markers and prognostic factors, and providing a powerful minimally-invasive approach for tumor progression assessment [6,7,8].

EVs, comprising exosomes (vesicles of endosomal origin) and microvesicles/microparticles (vesicles of plasma membrane origin) [9], have drawn attention as important players in a variety of diseases, and their role in oncology is currently an active area of research [10,11,12]. While exosomes are frequently considered 50–150 nm membrane vesicles, microvesicles are usually larger, with sizes up to 1 µm, being released by virtually all cell types to their surrounding environment [9,10,13]. Endothelial EVs in particular have been described as a hallmark of endothelial dysfunction and can be derived from endothelial cells (ECs) and in particular from brain microvascular ECs (BMECs) [14,15], the anatomical basis of the blood-brain barrier (BBB) [16]. Consequently, endothelial EVs share both cytoplasm content and cell surface markers with their parent cells, replicating their status [15,17]. Conditions such as hypoxia, irradiation, inflammation, oxidative stress-induced injury and shear stress can lead to the formation and release of endothelial EVs [9]. In BCBM formation, the interaction of BC cells (BCCs) with BMECs was shown to induce endothelial blebbing [18], a phenomenon that can be associated with endothelial EV release [19]. Thus, the BBB assumes a key role in BCBM, raising the hypothesis that it can constitute a source of biomarkers.

At the BBB, a high density of the glucose transporter 1 (GLUT1) is found in both luminal and abluminal membranes of BMECs [20]. Essentially all glucose transport at the BBB can be accounted for by the GLUT1, the gene of which is selectively expressed in the brain microvascular endothelium with nearly no expression in neurons or glial cells [21]. Besides GLUT1, the transferrin receptor (TfR or CD71) also appears to be exclusively expressed on BMECs and not on ECs lining the vessels in other tissues [22]. The TfR is responsible for the transport of iron into the brain parenchyma to assure iron homeostasis, vital for proper brain function [23]. As distinctive features, GLUT1- and TfR-positive endothelial EVs may comprise specific biomarkers released at the BBB level, and putatively increase in pathological conditions that compromise BBB integrity, as observed during BCCs extravasation in BCBM formation [24]. However, to our knowledge, such a hypothesis has never been tested.

Besides EVs, miRNAs have arisen as powerful and specific biomarkers for different types of cancer and metastasis, having unique expression profiles, depending not only on the type of cancer but also on the disease stage [8,25,26]. miRNAs are short noncoding RNAs of approximately 18–22 nucleotides that play a significant role in the regulation of multiple genes, modulating a plethora of cellular processes, including cell survival, apoptosis, carcinogenesis, and metastasis [27]. Therefore, dysregulation of miRNAs has a prominent role in cancer, and specific ones can be either upregulated or downregulated in different types of the disease, possibly functioning as oncogenes or as tumor suppressors [8,28]. Circulating miRNAs can be of special interest, due to their stability and easy quantification in biological fluids, allowing their usage in liquid biopsies for diagnosis and prognosis of oncological diseases [6,29]. Our previous next-generation sequencing (NGS) analysis of the miRNAome in a BCBM mouse model demonstrated that several miRNAs are deregulated in plasma prior to detection of brain metastases. Among such miRNAs are miR-194-5p and miR-802-5p, found to be downregulated, and miR-92a-1-5p, miR-205-5p, and miR-181a-1-3p, which were upregulated [30]. Therefore, such miRNAs can comprise valuable early biomarkers of BCBM formation, which remains uninvestigated.

Being aware of the urgent need to discover reliable and accessible diagnostic tools for BCBM, we aimed to discover biomarkers appearing in plasma with BCBM formation. We tested the hypothesis that BCBM formation would be associated with BBB endothelium-derived EVs release into the circulation and a dysregulation of specific miRNAs in plasma, and whether such alterations were related with distinctive phases of the brain metastases development process. An increase in TfR-positive EVs was found in plasma when BCBM were already established, but not prior to their detection, pointing to such EVs as a distinctive biomarker of advanced disease stages. Analysis of plasma samples by real-time quantitative PCR (RT-qPCR) corroborated miR-194-5p and miR-802-5p downregulation, as well as miR-92a-1-5p, miR-205-5p, and miR-181a-1-3p upregulation prior to BCBM detection. Moreover, analysis of brain sections by in situ hybridization (ISH) established the link between peripheral and brain events, which collectively point to such miRNAs as putative predictive biomarkers. In vitro studies in a BCBM formation model highlighted miR-194-5p and miR-205-5p as two of the circulating miRNAs expressed by BCCs, BMECs, and during BCC and BMEC interactions. Such miRNAs comprise valuable players in transendothelial migration of BCCs across the BBB, which points to their relevance as early biomarkers of the extravasation process.

## 2. Results

### 2.1. Circulating Brain Endothelial EVs Increase in Advanced Stages of BCBM

**Figure 1:**
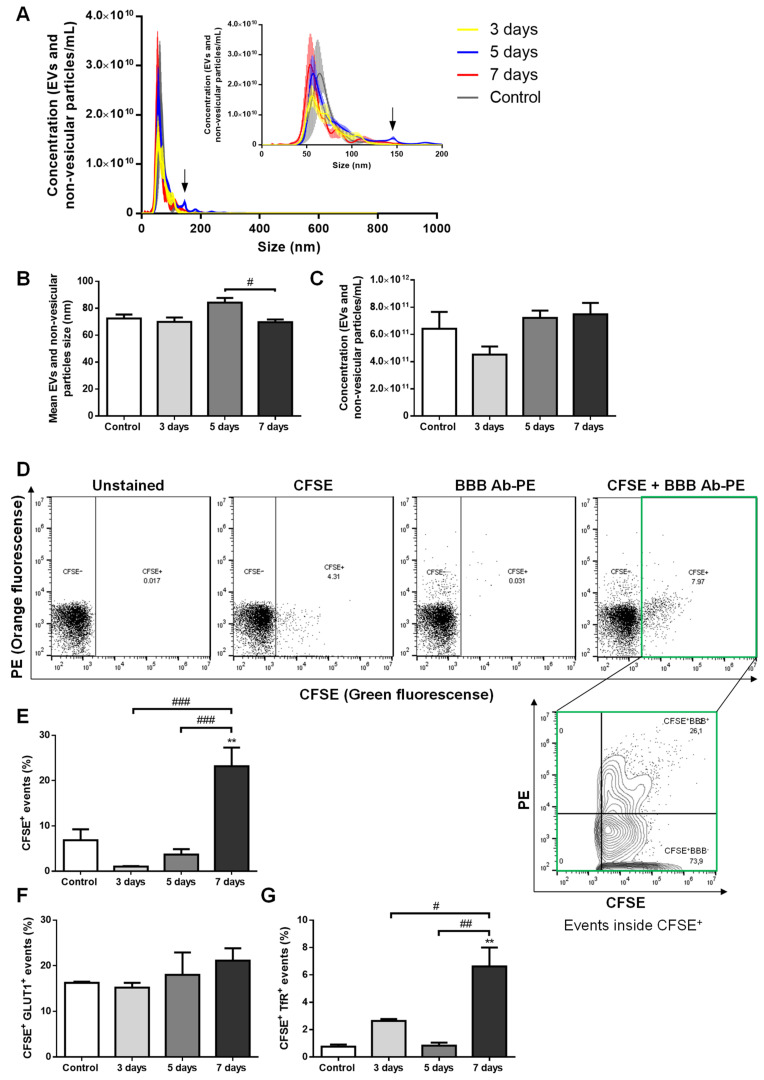
Extracellular vesicles (EVs) are released by brain microvascular endothelial cells in advanced stages of breast cancer brain metastases (BCBM) formation. 4T1 cells or vehicle (control) were inoculated into the carotid artery of female Balb/c mice, whole blood was collected upon sacrifice after 3, 5 and 7 days, and plasma was processed for analysis of EVs (comprising exosomes and microvesicles/microparticles) and non-vesicular particles. Nanoparticle tracking analysis (NTA) was performed to assess size distribution (**A**), mean size (**B**) and concentration (**C**), which revealed a sustained presence of exosome-like EVs and non-vesicular particles with BCBM formation, with an additional tiny peak observed at 5 days (arrow in **A**). EVs of brain endothelial origin were determined by flow cytometry using carboxyfluorescein diacetate succinimidyl ester (CFSE) to detect vesicular particles (CFSE^+^) and phycoerythin (PE)-conjugated antibodies against the blood-brain barrier (BBB) markers, glucose transporter 1 (GLUT1-PE) and transferrin receptor (TfR-PE), collectively referred to as BBB Ab-PE. Representative plots of unstained, only CFSE-labelled, only BBB Ab-PE-labelled, and CFSE+BBB Ab-PE-labelled plasma samples are presented, which allowed the quantification of vesicular particles (CFSE^+^) of brain endothelial origin (BBB^+^) inside the CFSE^+^ population (CFSE^+^ BBB^+^) (**D**). The percentage of vesicular particle (CFSE^+^) events increased at 7 days after 4T1 cells injection (**E**). No statistically significant differences were observed in CFSE^+^ GLUT1^+^ events within the CFSE^+^ population with BCBM (**F**), while CFSE^+^ TfR^+^ events within the CFSE^+^ population increased at 7 days (**G**). Statistical differences are denoted as ** *p* < 0.01 vs. control, and as # *p* < 0.05, ## *p* < 0.01 and ### *p* < 0.001 between indicated conditions by One-way ANOVA. Data represented are means ± SEM, *n* = 3.

In order to disclose the putative release of brain endothelial EVs to the circulation with BCBM formation, we used an established mouse model of the pathology. One of the most aggressive TN BCCs, the 4T1 cells [31], were inoculated into the common carotid artery of female Balb/c mice to direct the malignant cells to the brain and allow preferential brain metastases formation, which is observed 3 days after BCC injection [24,30]. Plasma samples were collected before and after metastases detection, as well as in vehicle-injected controls, and were analyzed for EV content, and their brain endothelial origin was inspected based on analysis of GLUT1^+^ and TfR^+^ events (Figure 1).

By nanoparticle tracking analysis (NTA), which detects EVs and non-vesicular particles (i.e., of inorganic origin), we studied the size distribution and content of such vesicles. We observed that plasma EVs and non-vesicular particles from the different time points essentially displayed a similar size distribution profile, with most of them presenting a size below 150 nm (Figure 1A), a feature described to correspond to exosome dimensions [10]. Concerning the vesicles mean size, no major differences were observed during the time of metastases development, apart from those at 5 days, which presented on average a higher diameter than at 7 days (*p* < 0.05, Figure 1B) and that can be explained by the appearance of a small peak of circa 150 nm in the size distribution profile (Figure 1A). Nevertheless, following the latest recommendations on minimal information for studies of extracellular vesicles (MISEV’s) [32], and to avoid potential sample misidentification, we decided to proceed with the reference to EVs throughout the manuscript. There were no significant temporal differences in EVs and non-vesicular particle concentrations, which ranged from 4 to 8 × 10^11^ particles/mL (Figure 1C).

Brain endothelial EV content in plasma with BCBM formation was assessed by flow cytometry. We employed the flow cytometry strategy for EV population analysis, which does not require EV isolation or concentration prior to staining but relies on the staining of vesicular particles with carboxyfluorescein diacetate succinimidyl ester (CFSE) previously described by us [33]. In each sample, the CFSE labelling allowed the quantification of the proportion of vesicular particles (CFSE^+^), distinguishing them from non-vesicular particles (CFSE^−^), and further evaluation of the events within the vesicular (CFSE^+^) population (Figure 1D). The percentage of CFSE^+^ events varied among the different samples, with 7 days plasma presenting the highest values (*p* < 0.01 vs. control, and *p* < 0.001 vs. 3 and 5 days, Figure 1E), indicating an increasing release of EVs in advanced stages of BCBM formation. Regarding CFSE^+^ GLUT1^+^ plasma EVs, an increase from ~16% in the control to ~21% in 7 days plasma samples was observed though not statistically significant (Figure 1F). Despite the lower amounts of CFSE^+^ TfR^+^ plasma EVs as compared with CSFE^+^ GLUT1^+^ ones, the number of EVs expressing the TfR increased, being higher at 7 days (*p* < 0.01 vs. control and 5 days, and *p* < 0.05 vs. 3 days, Figure 1G), reflecting an increase in endothelial EVs of BBB origin.

Overall, though no early (3 or 5 days) increase in EVs was observed, our results revealed augmented vesicular (CFSE^+^) and CFSE^+^ TfR^+^ EVs in the circulation when BCBM are already established (7 days), supporting our hypothesis that BBB-derived EVs can comprise a relevant biomarker of the pathology, particularly at later stages.

### 2.2. Circulating miRNAs Alterations Are Related with Their Brain Parenchyma Deregulation

**Figure 2:**
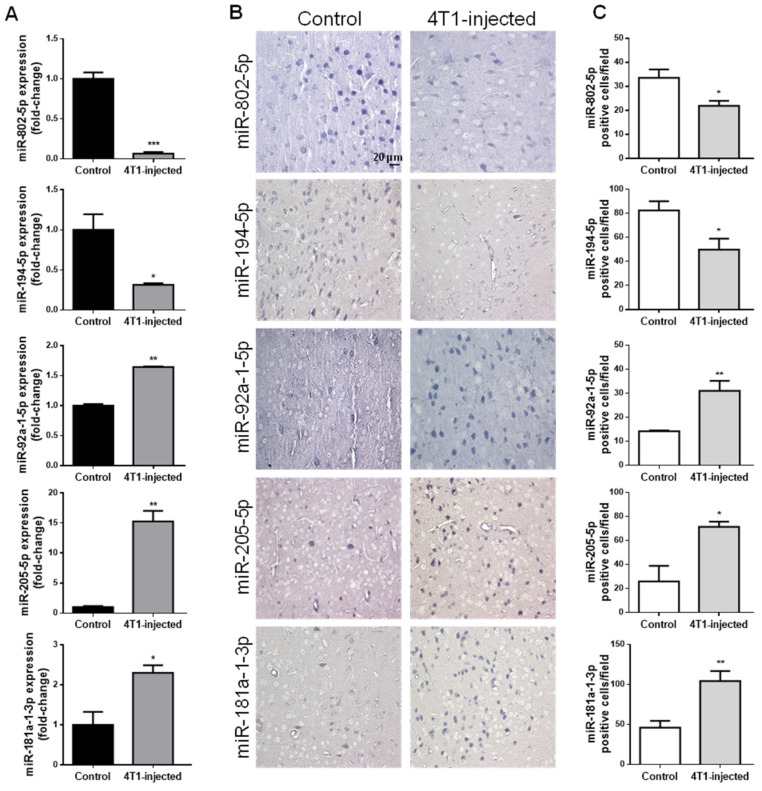
Circulating miRNAs alterations are related with their brain parenchyma deregulation in early stages of breast cancer brain metastases formation. 4T1 cells or vehicle (control) were inoculated into the carotid artery of female Balb/c mice and after 3 days, whole blood was collected, and brains harvested after sacrifice. Plasma was processed for real-time quantitative PCR (RT-qPCR) and sections of cranial hippocampus were processed for in situ hybridization (ISH) analysis of the different miRNAs. RT-qPCR analysis highlighted the downregulation of miR-802-5p and miR-194-5p, and the up-regulation of miR-92a-1-5p, miR-205-5p and miR-181a-1-3p in 4T1-injected mouse plasma, with the results presented as fold-change vs. control (**A**). ISH analysis validated the same miRNAs downregulation and upregulation, as shown by the expression of these miRNAs in bluish stained brain cells (**B**), and ascertained by semi-quantitative analysis of the number of each miRNA positive cell per field (**C**). Statistical differences are denoted as * *p* < 0.05, ** *p* < 0.01, *** *p* < 0.001 vs. control by two-tailed unpaired Student’s *t*-test. Data represented are means ± SEM, *n* = 3.

In addition to circulating EVs, circulating miRNAs can also comprise valuable tools as potential disease biomarkers. In that regard, miRNAs previously described to be altered prior to BCBM detection by NGS [30] were studied in mouse plasma samples and in hippocampal sections of vehicle- and 4T1-injected mice 3 days after inoculation, a time point at which BCBM are not yet patent (Figure 2).

By RT-qPCR analysis of plasma samples, we confirmed that miRNAs previously observed to be downregulated (miR-802-5p and miR-194-5p) were indeed decreased 3 days after 4T1-injection; on the other hand, miRNAs reported to be upregulated (miR-92a-1-5p, miR-205-5p and miR-181a-1-3p) were increased in 4T1-injected animals (Figure 2A), and this validated previous data ensuing from NGS analysis [30].

To establish whether the aberrant expression of these five miRNAs in plasma is associated with a corresponding deregulation in the brain, we performed ISH of brain sections at the same timepoint (Figure 2B) and further performed the semi-quantitative analysis of the number of positive cells for each miRNA (Figure 2C). We observed that brain resident cells in normal conditions (control) expressed both miR-802-5p and miR-194-5p and that the number of miR-802-5p-positive cells and miR-194-5p-positive cells significantly decreased comparatively to control in 4T1-injected mice (*p* < 0.05), in accordance with the RT-qPCR data. On the other hand, ISH revealed that miR-92a-1-5p, miR-205-5p and miR-181a-1-3p expression increased in 4T1-injected mice, an increase supported by the semi-quantitative analysis of each miRNA-positive cell (*p* < 0.01 for miR-92a-1-5p and miR-181a-1-3p; and *p* < 0.05 for miR-205-5p). Interestingly, miR-802-5p downregulation was observed throughout BCBM formation, while miR-92a-1-5p upregulation was only significant after 3 days (i.e., prior to BCBM detection, Figure S1).

Collectively, the present results obtained by RT-qPCR analysis reinforce the previously obtained indications of deregulated miRNAs in early phases of brain metastases formation [30]. Moreover, the parallelism established for the first time between plasma and brain alterations suggest that peripheral alterations are related with those occurring in the brain with the formation of BCBM. Thus, miR-802-5p, miR-194-5p, miR-92a-1-5p, miR-205-5p and miR-181a-1-3p appear as strong candidates as early biomarkers of BCBM.

### 2.3. miRNAs Are Differentially Released by BCCs, BMECs and During BCCs–BMECs Interaction

**Figure 3:**
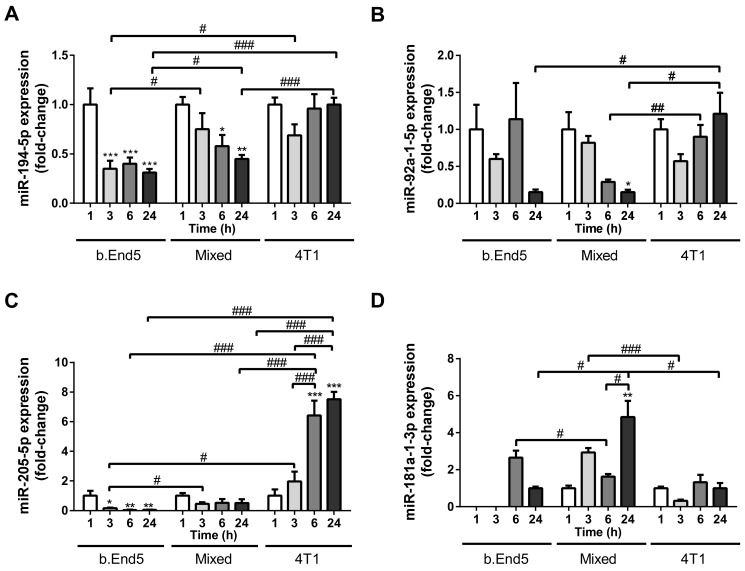
Differential release of miRNAs by single cultures of brain microvascular endothelial cells (BMECs) or breast cancer cells (BCCs), and mixed BMEC–BCC cultures. Single cultures of 4T1 cells (BCCs), b.End5 cells (BMECs) and mixed cultures of b.End5 and 4T1 (previously labelled with CellTracker™ Green) cells, were performed under physiological shear stress for 1, 3, 6 and 24 h, after which the media were collected and processed for real time quantitative PCR of the indicated miRNAs. The results are presented as fold-change vs. 1 h of each culture system (with the exception of miR-181a-1-3p in b.End5 single cultures, which were normalized for 24 h), and highlight significant alterations with time and between cultures for miR-194-5p (**A**), miR-92a-1-5p (**B**), miR-205-5p (**C**) and miR-181a-1-3p (**D**). Statistical differences are denoted as * *p* < 0.05, ** *p* < 0.01, *** *p* < 0.001 vs. 1 h of each culture, and by # *p* < 0.05, ## *p* < 0.01, ### *p* < 0.001 between indicated conditions, determined by one-way ANOVA within each culture against time, and by two-tailed unpaired Student’s *t*-test for each time point between cultures. Data represented are means ± SEM, *n* = 3.

Having established the circulating miRNAs potential as early biomarkers of BCBM formation, we aimed to elucidate BCCs, BMECs and BCC–BMEC interaction contribution to their release. For that, cell culture media of mixed cultures of 4T1 and b.End5 cells, as well as of their respective BCC and BMEC single cultures used as controls, were evaluated by RT-qPCR against time for each of the studied circulating miRNAs (Figure 3).

Regarding the downregulated miRNAs in plasma (miR-802-5p and miR-194-5p), we detected no miR-802-5p expression in any of the cell culture media analyzed, indicating that neither BCCs, nor BMECs, are responsible for its secretion. On the contrary, both b.End5 and 4T1 cells secreted miR-194-5p into the cell media, with decreasing secretion observed over time in b.End5 cultures (*p* < 0.001 3/6/24 h vs. 1 h) and in mixed cultures (*p* < 0.01 24 h vs. 1 h, *p* < 0.05 6 h vs. 1 h, Figure 3A). Interestingly, the lowest miR-194-5p release was observed in b.End5 (*p* < 0.05 at 3 h and 24 h vs. mixed culture), and lower values were observed in mixed cultures as compared with 4T1 single cultures (*p* < 0.001 at 24 h), suggesting that b.End5 cells account for the downregulation of this miRNA.

Regarding the three miRNAs found upregulated in plasma (miR-92a-1-5p, miR-205-5p and miR-181a-1-3p), we observed their release in all in vitro conditions. A time-dependent decrease in miR-92a-5p in mixed cultures was observed, with values lower than those observed in 4T1 at prolonged incubation times (*p* < 0.01 at 6 h and *p* < 0.05 at 24 h, Figure 3B). Such downregulation in mixed cultures, in opposite to the upregulation in plasma, suggest that other cell types are responsible for the overexpression observed in vivo. The most notable observation about miR-205-5p was its remarkable increase over time in 4T1 cultures (*p* < 0.001 at 6 and 24 h vs. 1 and 3 h) and its significantly higher fold change variations than in b.End5 and mixed cultures (Figure 3C). These findings suggest that 4T1 cells are the ones responsible for the augmented release of miR-205-5p. Regarding miR-181a-1-3p, the most notable observation was the upregulation in mixed cultures at 24 h, as compared with each single culture (*p* < 0.05, Figure 3D). This finding raises the possibility that the interaction between BMECs and BCCs accounts for miR-181a-1-3p overexpression.

From these in vitro studies, we can conclude that miR-194-5p, miR-92a-1-5p, miR-205-5p and miR-181a-1-3p are released by BCCs, BMECs and/or during BCC–BMEC crosstalk, in contrast with miR-802-5p that is not released in these cellular systems. These observations, taken together with the miRNAome observed in in vivo studies, suggest that cell types other than BCCs or BMECs are responsible for the downregulation of miR-802-5p and upregulation of miR-92a-1-5p observed in plasma Moreover, they suggest that the overexpression of miR-181-1-3p results from the interaction between BCCs and BMECs, and that BMECs contribute to the downregulation of miR-194-5p, while BCCs account for the upregulation of miR-205-5p.

### 2.4. miR-194-5p and miR-205-5p Release Reflect Their Cellular Expression

**Figure 4:**
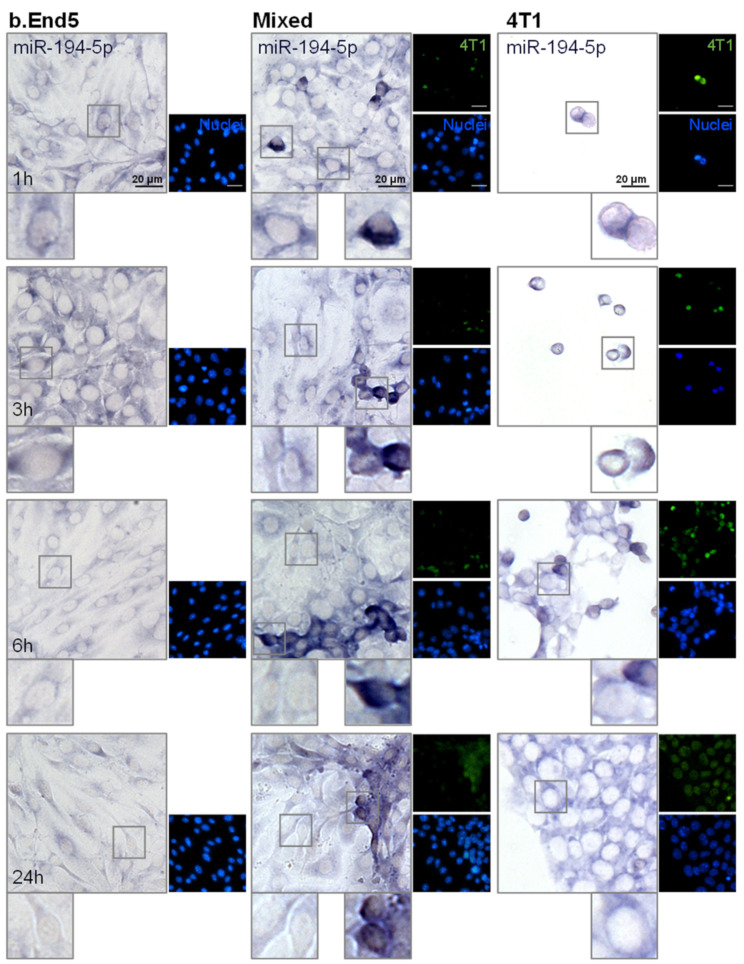
miR-194-5p is expressed by brain microvascular endothelial cells (BMECs), breast cancer cells (BCCs) and during BMEC–BCC interaction. Single cultures of 4T1 cells (BCCs), b.End5 cells (BMECs) and mixed cultures of b.End5 and 4T1 cells (previously labelled with CellTracker™ Green) were performed under physiological shear stress for 1, 3, 6 and 24 h, after which cells were fixed and processed for in situ hybridization (ISH) for miR-194-5p and nuclei (blue) were counterstained with Hoechst 33342. Insets highlight major cellular alterations of miRNA expression in both cell populations (BMECs: left; BCCs: right) over time. ISH for miR-194-5p revealed a bluish coloration in BMECs, BCCs and particularly in BCCs interacting with BMECs.

**Figure 5:**
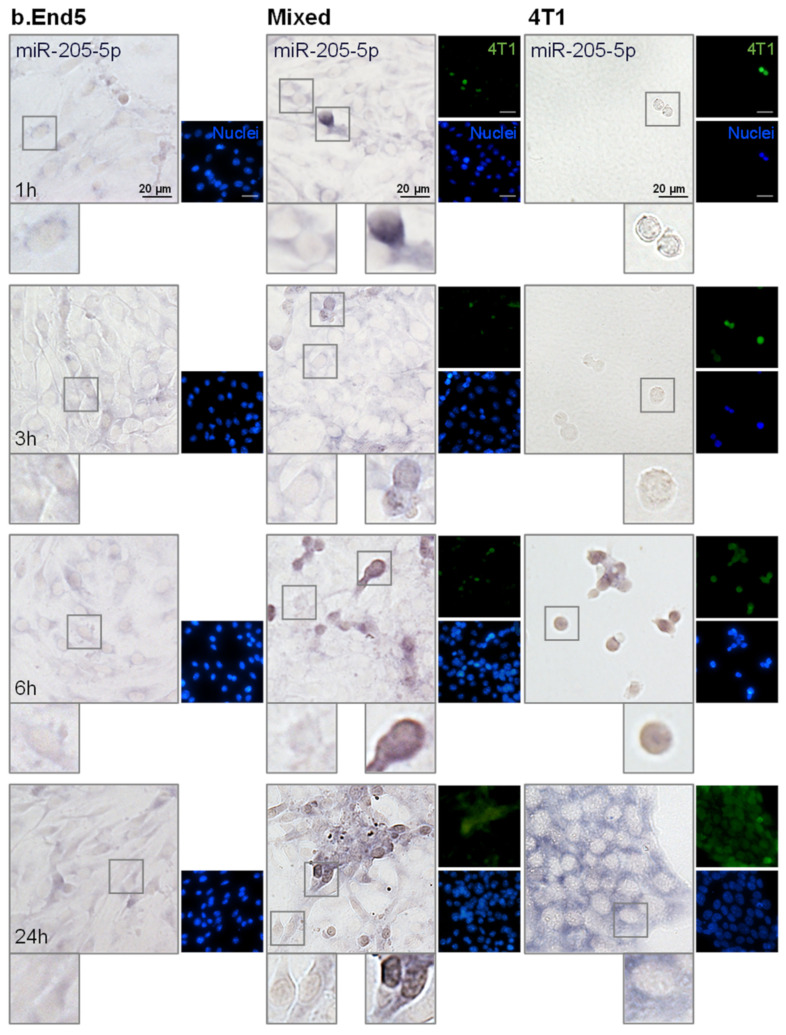
miR-205-5p is expressed by brain microvascular endothelial cells (BMECs), breast cancer cells (BCCs) and during BMEC–BCC interaction. Single cultures of 4T1 cells (BCCs), b.End5 cells (BMECs) and mixed cultures of b.End5 and 4T1 cells (previously labelled with CellTracker™ Green) were performed under physiological shear stress for 1, 3, 6 and 24 h, after which cells were fixed and processed for in situ hybridization (ISH) for miR-205-5p and nuclei (blue) were counterstained with Hoechst 33342. Insets highlight major cellular alterations of miRNA expression in both cell populations (BMECs: left; BCCs: right) along time. ISH for miR-205-5p revealed a bluish coloration mainly in large BCCs clusters at later time points.

Having evaluated miRNAs release in vitro, the two most promising circulating miRNAs, the downregulated miR-194-5p and the upregulated miR-205-5p, were further analyzed in each of the studied populations, BMECs and BCCs, to ascertain their origin. For that, ISH assays were performed in single cultures of b.End5 and of 4T1 cells, as well as in mixed b.End5–4T1 cells, taking advantage of CellTracker™ Green-labelled 4T1 cells to distinguish both populations (Figure 4 and Figure 5, respectively).

We observed that miR-194-5p is expressed by b.End5 cells and by 4T1 cells, in single and in mixed cultures (Figure 4), as indicated by the observed bluish staining. In b.End5 cells, the expression of miR-194-5p appeared to decrease at later time points (6 and 24 h) in both single and mixed cultures, whereas this miRNA expression is particularly evident in 4T1 cells alone and in ‘metastasis-like’ clusters, preferentially locating in the perinuclear region of the cells. Interestingly, b.End5–4T1 prolonged interaction led to a notably decreased expression of miR-194-5p in 4T1 cells (from 6 to 24 h), possibly a consequence of this miRNA downregulation caused by b.End5 and in line with the sustained decrease observed in released miR-194-5p in cultures and with the lower levels observed in plasma.

Regarding miR-205-5p, we observed that it is slightly expressed by b.End5 cells, but mainly by 4T1 cells, both in single and in mixed cultures (Figure 5). Moreover, as evidenced by the cytoplasmatic light blue staining, miR-205-5p is less expressed by BMEC and BCCs than miR-194-5p up to 6 h. In contrast, at 24 h a notable bluish staining was observed in the cytoplasm in large 4T1 clusters in single culture, corresponding to a clear expression of this miRNA, probably associated with the correspondingly higher content observed in the cell medium of 4T1 cells in single culture. Thus, ISH results corroborate previous RT-qPCR data from cell media, which pointed to 4T1 cells as the ones responsible for miR-205-5p production.

Collectively, we conclude that miR-194-5p and miR-205-5p are released and expressed by BCCs, BMECs and with BCC–BMEC interaction, at least partially reflecting miRNA alterations found in the circulation.

## 3. Discussion

Brain metastases such as those derived from TNBC are the ones presenting the highest mortality and the lowest survival rates. Besides effective targeted treatments, a good biomarker for a robust diagnosis would have the potential to increase patients’ survival and life quality. In this work, we provide evidence pointing to BBB-derived EVs as candidate biomarkers of the disease at advanced stages, as well as to circulating miRNAs with a deregulated expression prior to metastasis detection, as putative early biomarkers of the disease.

Tumor biomarker is a term used to describe a potential indicator of cancer development and progression specific to a particular cancer type, like proteins, nucleic acids (DNA, mRNA, miRNA), and EVs [25,34]. Ideally, a new biomarker should fulfill an unmet need in cancer detection; alternatively, it should provide advantages over preexisting biomarker(s), such as being more accurate and simpler to measure, faster, or cheaper [35]. Here, we employed a flow cytometry methodology recently established by our team, which enables population analysis of EVs in samples characterized by challenging small volumes while reducing overall sample processing time [33]. Such methodology has the advantage of not requiring EV isolation or concentration prior to staining, enabling the analysis of EVs in both purified and non-purified biological samples. Profiting from such a state-of-the-art approach, we were able to assess BBB-derived EVs during BCBM development in a reliable mouse model [24,30].

In the past decades, EVs have emerged as key players in cancer biology and as powerful tools to ascertain disease severity and progression [10,12,36]. In that regard, BBB-specific EVs in body fluids, released into the circulation from luminal membranes of BMECs and potentially shuttled across the BBB from the abluminal side, could correspond to biomarkers useful for the diagnosis or monitoring of brain diseases. Indeed, Haqqani and colleagues have shown that circa 20% of the BMEC-EV proteins were absent in exosomes from other cell types [37]. As virtually all cells release EVs [10], we hypothesized that EVs derived from BMECs found in the circulation could comprise a distinctive biomarker of BCBM formation, putatively reflecting disease pathobiology in which BBB disruption has been reported [24]. Limited data regarding EVs arising from the BBB exists, and consequently, there is a critical need to better understand BBB-derived EV variations with disease progression. 

It is acknowledged that BBB-derived EVs can potentially constitute communication tools among different cell types, able to cross the cells, and playing critical roles in physiological and pathological processes [37,38]. Importantly, BBB-derived EVs may contain BBB-specific molecules, like vascular endothelial (VE)-cadherin or platelet endothelial cell adhesion molecule [9], and receptors such as insulin receptor, low-density lipoprotein receptor, and TfR [37]. Patients with metastatic BC or with large BC tumors presented higher VE-cadherin-positive endothelial EVs in plasma compared with those with small tumors, pointing to a possible association with endothelial activation in vivo [39]. Our flow cytometry analysis revealed that 7 days after the injection of BCCs, when metastases are already established, CFSE^+^ EVs are increased. Accordingly, elevated levels of plasma EVs were observed in BC patients as compared to controls, and were shown to correlate with disease stage and severity [36]. Furthermore, elevation of circulating VE-cadherin-positive endothelial EVs in metastatic BC has been reported [40], pointing to BBB-derived EVs potential as a biomarker of BCBM. Interestingly, GLUT1 was also identified in EVs as a potential biomarker derived from metastatic BCCs that may be used for disease diagnosis and prognosis [41]. Surprisingly, in the present study no statistically significant alterations were observed in GLUT1^+^ EV levels in the circulation. Although GLUT1 is mainly abundant in BMECs, it is also expressed in erythrocytes [42] and by BCCs, in primary tumors and in distant metastases [43,44], which may contribute to GLUT1^+^ EVs release even in basal conditions. Compared to GLUT1, TfR has also been shown to be mainly expressed by BMECs [22], though not exclusively [45]. Indeed, iron uptake mediated by TfR is also a determinant for highly proliferative cells such as those in breast tumors, comprising a marker of poor prognosis in BC patients [46]. Nevertheless, it has been demonstrated that TfR-positive endothelial EVs can be obtained from BMECs, comprising a putative BMEC-specific molecular signature useful in health and disease states [37]. Although not comprising an early biomarker of the disease, we can suggest that endothelial TfR^+^ EVs, which significantly increased at 7 days, can comprise a distinctive indicator of the disease at an advanced stage of brain metastases development.

Current evidence suggests that EVs contain approximately half of circulating RNAs in plasma [47,48]. In that regard, the detection of a specific miRNA signature could give the possibility of a more robust early diagnosis than the sole analysis of BBB-derived EVs. miRNA expression patterns can be associated not only with BC subtypes but also with disease progression/stage and overall survival [49,50,51]. Recently, we observed the deregulation of several miRNAs in plasma with BCBM development, including at an initial stage, where metastases were still not detected [30]. This discovery prompted us to disclose their putative potential as early peripheral biomarkers of brain metastases development. We corroborated the existence of altered miRNAs levels in plasma and further showed the corresponding changes in their brain expression. Positive correlations between circulating miRNAs and tissue expression in BC patients have already been reported for several miRNAs, including miR-145 and miR-451 [52], miR-106a-5p and miR-20b-5p [53], miR-182 [54], and miR-139 [55], while for miR-195 [51] and miR-181a [56], a negative correlation was found. In our model, we observed that miR-802-5p and miR-194-5p downregulation in plasma is also observed in the brain, whilst miR-92a-1-5p, miR-205-5p and miR-181a-1-3p are upregulated in plasma as well as in the brain. Accordingly, miR-802-5p was shown to have a lower expression in BCCs compared to normal breast epithelial cells, decreasing with BC proliferation [57], underscoring its putative tumor suppressive role in the metastatic progression. Our data support such observations, as a sustained decrease in expression of miR-802-5p in brain parenchyma over time was observed. On the other hand, decreased levels of miR-92a have been associated with tumor size and a positive lymph node status in BC patients [58]. However, such reported alterations do not correspond to an early disease state but to an advanced one, and indeed in our model, miR-92a-1-5p overexpression was observed only at an early time point, prior to metastasis detection. To our knowledge, this is the first work associating these early miRNA brain alterations and plasma levels in the scope of TN BCBM formation in vivo.

In order to validate the deregulation of miRNAs observed in plasma samples, to establish whether they are produced by BBB ECs and/or BCCs, and to ascertain the reflex of cellular crosstalk on their production and release, we employed an in vitro model of BCBM formation using the same TN BCCs (4T1 cells), in combination with an improved BBB mouse model incorporating shear stress [59]. With the exception of miR-802-5p, we confirmed the release into the extracellular medium of all the studied miRNAs by b.End5 and/or by 4T1 cells. Of course, we cannot discard in vivo brain complexity and other brain resident cells contribution to the system; nevertheless, our results point to miR-194-5p, miR-92a-1-5p, miR-205-5p and miR-181a-1-3p appearing to be regulated, at least partially, by BMECs, BCCs and during BMEC-BCC interaction. A recent study demonstrated that miR-183-5p is expressed by 4T1 cells and transferred to macrophages, promoting proinflammatory cytokine secretion, and contributing to BC progression [60]. On the other hand, miR-200c is downregulated in TN BCC lines, such as 4T1 and MDA-MB-231, as well as in clinical specimens, being associated with poor patient overall survival and disease-free survival [61]. Importantly, TNBC-derived EVs, carrying miR-181c, promoted BBB breakdown [62]. The same was observed for miR-105, detected in the circulation at the premetastatic stage, which efficiently destroyed tight junctions, compromising endothelial integrity and promoting BCBM [63]. Also, BC-secreted miR-939 predicts worse prognosis in TNBCs, downregulating VE-cadherin in endothelial cells and enhancing the transendothelial migration of BCCs [64]. Likewise, we may suggest that highly secreted miRNAs by 4T1, like miR-205-5p, can ultimately contribute to the impairment of BBB properties, facilitating BCC extravasation and BCBM establishment.

Several studies of different types of human cancers have led to controversial results concerning deregulation of miR-205-5p expression. In fact, miR-205-5p expression was described to be decreased in BC [49,65], and its downregulation was suggested to play a significant role in TNBC metastasis, promoting cell migration and EMT, comprising a potential diagnostic or therapeutic target [66]. In contrast, upregulation of miR-205-5p in cervical, lung, and renal cancer cell lines under hypoxic conditions, was shown to promote epithelial-mesenchymal transition (EMT) [67]. In the present work, we observed miR-205-5p upregulation in cell cultures, particularly in 4T1 cells, in line with its overexpression observed in vivo and previously reported by us [30]. Thus, the observed miR-205-5p upregulation can comprise a mechanism contributing to 4T1 cells extravasation (i.e., 3 days post-injection, [24]). Such upregulation may be followed by a later downregulation when BCBM is already established, in line with the downregulation reported in TNBC patients [49].

An association between in vitro and in vivo release/expression of miR-194-5p was observed, with a downregulation of miR-194-5p in 4T1 cells upon prolonged BMEC–BCC contact. Contradictory results regarding a putative oncogenic/tumor suppressor role of miR-194-5p have been reported. In fact, miR-194-5p has been described to be downregulated in Wilms tumor, a nephroblastoma, where it was also shown that miR-194-5p acts as a tumor suppressor in nephroblastoma cells via downregulation of EMT, modulating tumor cell migration and invasion [68]. As far as BC is concerned, the inhibition of miR-194-5p in MCF7 cells was shown to correlate with decreased proliferation, migration and tumor growth [69], while in HER2 overexpressing BCCs, miR-194 knockdown promoted cancer cell migration and invasion [70]. Mao et al. reported that the overexpression of miR-194-5p in both MCF7 and MDA-MB-231 cells promoted BCC proliferation, migration and invasion, while repressing cell death [71]. In accordance, in our in vitro system, 4T1 cells alone and in mixed culture, released and expressed miR-194-5p, especially in ‘metastasis-like’ clusters, being particularly evident in more marginal, possibly more invasive/migratory cells. Importantly, BMECs appear to contribute to miR-194-5p downregulation, as observed by RT-qPCR data and ISH analysis. To our knowledge, miR-194-5p alterations upon BCC-BMEC crosstalk have not yet been studied. Therefore, our results may constitute the first report presenting miR-194-5p regulation in a robust in vitro model of BCBM. These findings, recapitulating the in vivo observations reported here and previously [30], point to miR-194-5p as a putative biomarker of the disease and a target to be tackled.

The present study establish TfR-positive EVs and downregulated (miR-194-5p and miR-802-5p) and upregulated (miR-92a-1-5p, miR-205-5p, and miR-181a-1-3p) miRNAs as candidates for biomarkers of BCBM development, deserving to be translated into the clinical practice. Therefore, it would be important to monitor a cohort of BC patients after treatment of the primary tumor until the development of brain metastases regarding the plasma levels of these biomarker candidates, and to establish their relationship with the patients’ neurologic status and brain imaging. This would allow the identification of the most robust biomarkers of the development of brain metastases, according to the disease stage, which could be implemented in the clinic for a timely detection of patients deserving therapeutic intervention.

## 4. Materials and Methods

### 4.1. Mouse Model of BCBM

**Figure 6:**
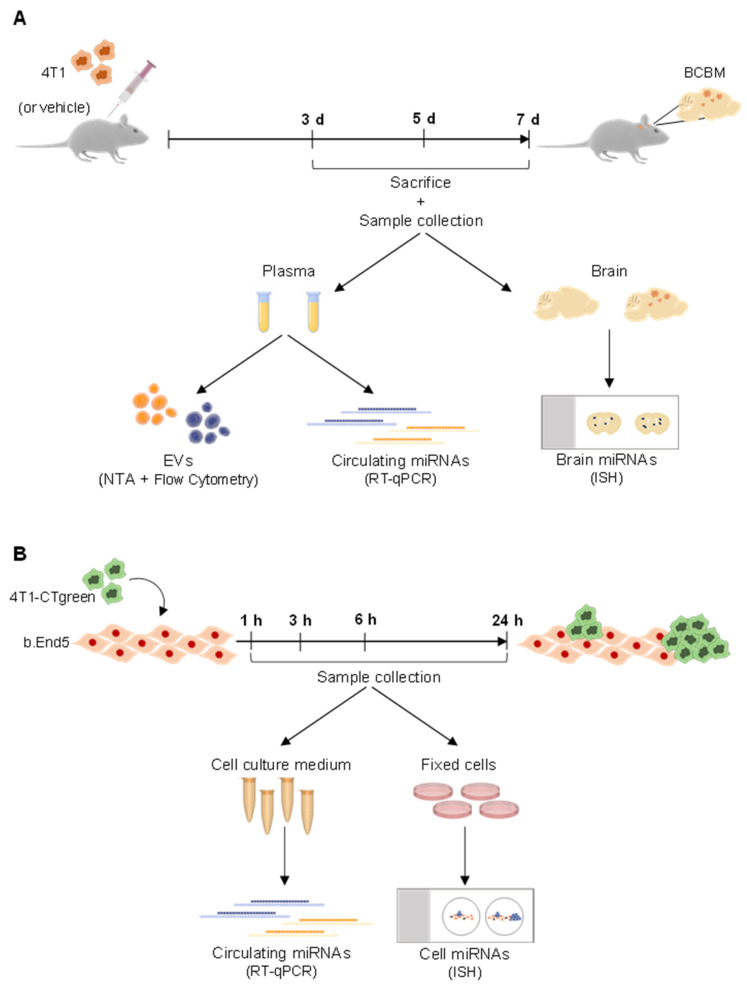
Schematic representation of the experimental design employed regarding in vivo and in vitro studies, as well as sample collection and the following procedures. (**A**) A mouse model of breast cancer brain metastases development was used, relying on the inoculation of murine mammary carcinoma triple negative 4T1 cells, or vehicle (control), into the carotid artery of Balb/c mice. Plasma was collected on the day of sacrifice, performed after 3, 5, and 7 days (d) post-inoculation. Plasma samples were used for quantification of extracellular vesicles (EVs) by Nanoparticle Tracking Analysis (NTA) and by flow cytometry, as well as for early (i.e., 3 days) evaluation of circulating miRNAs by real time quantitative PCR (RT-qPCR). After 3 days post-inoculation, brains were also harvested for analysis of miRNAs by in situ hybridization (ISH). (**B**) An in vitro mouse model of BCBM formation was also used. The mouse endothelioma cell line (b.End5) was cultured for 48 h to allow the formation of a confluent monolayer, after which physiological shear stress was applied. 24 h later, a mixed culture was initiated by seeding of 4T1 cells previously labelled with CellTracker™ Green CMFDA Dye (4T1-CTgreen) onto b.End5 monolayers. Single cultures were run in parallel as controls. Cell cultures were maintained for 1, 3, 6 and 24 h time points, after which culture media were collected for RT-qPCR analysis of released miRNAs and cells were fixed for ISH analysis.

A mouse model of BCBM was used, relying on the inoculation of murine mammary carcinoma TN 4T1 cells in Balb/c mice, implemented by the team [24,30]. The 4T1 cells were maintained in RPMI 1640 medium (PAN Biotech, Aidenbach, Germany) supplemented with ultra-glutamine I (Lonza, Basel, Switzerland) and 5% heat-inactivated fetal bovine serum (FBS, PAN Biotech) in a 5% CO_2_ atmosphere at 37 °C. BCCs (1 × 10^6^ 4T1 cells in 200 µL of Ringer-HEPES) were xenografted, under isoflurane anesthesia, in the right common carotid artery of 7-8-week-old female Balb/c mice (Charles River Laboratories, Wilmington, MA, USA). Control mice were inoculated with Ringer-HEPES. Mice were housed and bred in the animal facility of the Biological Research Centre, Szeged, Hungary. Blood samples were collected after 3, 5, and 7 days post-inoculation, and brains were harvested after 3 days post-inoculation (experimental layout in Figure 6).

All animal experimentation was performed by certified team members at the Biological Research Centre, according to the recommendations of the Declaration of Helsinki and Tokyo, and were performed according to the EU Directive 2010/63/EU on the protection of animals used for experimental and other scientific purposes. The protocol was reviewed and approved by the Regional Animal Health and Food Control Station of Csongrád County (license numbers: XVI./2980/2012 and XVI./764/2018).

### 4.2. Cell Model of BCBM

As an in vitro model that mimics BCBM development, mixed cultures of mouse Balb/c brain endothelioma cell line, b.End5, and 4T1 cells were used. b.End5 cells were maintained in Dulbecco’s modified Eagle’s medium (DMEM, Gibco, Life Technologies, New York, NY, USA) supplemented with 10% FBS, 1% non-essential amino acids (Biochrom AG, Berlin, Germany), 2 mM L-glutamine (Biochrom), 1mM sodium pyruvate (Biochrom) and 1% antibiotic-antimycotic solution (Sigma Aldrich, St. Louis, MO, USA) in a 5% CO_2_ atmosphere at 37 °C. b.End5 cells (5 × 10^4^ cells/mL) were plated onto glass coverslips previously coated with rat tail collagen I (Corning, New York, NY, USA) at 50 μg/mL. After 48 h, laminar non-pulsatile physiologic shear stress (1.5 dyn/cm^2^) was applied for 24 h, as implemented in our lab [59]. In order to distinguish both cell populations, 4T1 cells were labelled with CellTracker™ Green CMFDA Dye (2.5 μM; Thermo Fisher Scientific, Waltham, MA, USA), and then applied (1 × 10^5^ cells/mL) in DMEM on top of b.End5 monolayers. Mixed cultures and corresponding single cultures (controls) were kept on shear stress conditions for 1, 3, 6 and 24 h, time points after which cell culture media were collected and cells were fixed with 4% paraformaldehyde (PFA, Sigma-Aldrich) in phosphate-buffered saline (PBS) for 20 min at room temperature (experimental layout in Figure 6).

### 4.3. Collection of Plasma

Approximately 500 µL of blood samples were collected directly from the heart of live mice under isoflurane anesthesia. The samples were collected using syringes previously washed with ethylenediaminetetraacetic acid (EDTA, 0.5 M, pH 8.0) into tubes containing 40 µL of the anticoagulant. After collection, blood samples were centrifuged for 15 min at 1500× *g*, followed by 2 min at 13,000× *g* to obtain platelet-free plasma. Samples were stored at −80 °C until further analysis.

### 4.4. Collection of Brains

Anesthetized mice were perfused with PBS, followed by tissue fixation with 4% PFA in PBS. Brains were harvested, post-fixed overnight in 4% PFA in PBS at 4 °C and were kept in PBS containing 0.1% sodium azide. Cranial hippocampus (coronal sections at −1.82 mm Bregma coordinate), the most affected brain region in terms of BCBM formation in this animal model [30], were paraffin embedded and cut into 6-μm-thick sections.

### 4.5. Nanoparticle Tracking

All plasma samples were analyzed by the NS300 NTA system with red laser (638 nm) (NanoSight—Malvern Panalytical, Malvern, UK), which detects vesicular (EVs) and non-vesicular particles (i.e., of inorganic origin). Samples were pre-diluted in filtered PBS to achieve a concentration within the range for optimal NTA analysis of EVs and non-vesicular particles concentration and size distribution. Video acquisitions were performed using a camera level of 16 and a threshold between 5 and 7. Five to nine videos of 30 s were captured per sample. Analysis of particle concentration per mL and size distribution were performed with the NTA software v3.4.

### 4.6. EVs Staining and Preparation

For staining, 2 × 10^9^ EVs and non-vesicular particles of each plasma sample were diluted in 40 µL of filtered PBS containing 4 µg of anti-GLUT1 conjugated to PE (Abcam, #ab209449, Cambridge, UK) or containing 4 µg of anti-CD71 conjugated to PE (Sysmex, #AY361032, Görlitz, Germany) and incubated for 1 h at 37 °C. Samples were incubated with CFSE (Thermo Fisher Scientific, #LTI C34554, Waltham, MA, USA) to distinguish vesicular (CFSE^+^) from non-vesicular particles (CFSE^−^), in a final concentration of 25.6 µM for 90 min at 37 °C. The staining with CFSE was done after the addition of the antibody to ensure that the dye would not interfere with the antibody staining. For removal of unbound CFSE and antibody, size exclusion chromatography (SEC) columns (iZON qEV original columns SP1, Oxford, UK) were used. Samples containing stained EVs, and appropriate controls (unstained and FMOs), were diluted up to 500 µL with filtered PBS and processed by qEV following manufacturer’s instructions. EV-enriched fractions #7, #8, and #9 (500 µL each) were pooled and retrieved for analysis by flow cytometry.

### 4.7. Flow Cytometry

For tracking of labeled EVs, flow cytometry of EV-enriched fractions was performed on an Apogee A60-Micro-Plus (Apogee Flow Systems, Hertfordshire, UK). As internal controls and before each FC experiment, commercially available mixes of beads (Apogee 1493 and Apogee 1517, Apogee Flow Systems, Hertfordshire, UK) were used as reference particles for the EVs detection. All samples were run at a flow rate of 1.5 µL/min using a 405 nm—LALS threshold of 70. Samples were acquired until a minimum of 200,000 events was reached. The acquired data was exported and analyzed with FlowJo software v10.4.2 (FlowJo LLC, Ashland, OR, USA).

### 4.8. RT-qPCR

Total RNA from plasma samples and cell media was firstly isolated using the miRNeasy Serum/Plasma Advanced Kit (Qiagen, Dusseldorf, Germany), according to the manufacturer’s instructions.

RNA was then transcribed into cDNA, using the miRCURY LNA RT Kit (Qiagen, Dusseldorf, Germany), according to manufacturer’s instructions, though the RNA volume used was increased four times, as previously optimized [30]. Prior to the reverse transcription reaction, the synthetic RNA UniSp6 RNA spike-in (Qiagen, Dusseldorf, Germany) was added to the mixture. The reaction was performed on a Biometra T-Combi thermocycler (Analytic Jena, Jena, Germany), using the following conditions: 42 °C for 60 min; 95 °C for 5 min to heat-inactivate the reverse transcriptase, and cooling down and storage at 4 °C.

RT-qPCR was performed using QuantStudio™ 7 Flex Real-Time PCR System (Applied Biosystems, Waltham, MA, USA) and miRCURY LNA SYBR Green PCR Kit (Qiagen, Dusseldorf, Germany) according to the manufacturer’s instructions using cDNA diluted at 1:6. The following conditions were used: 50 cycles of 95 °C for 15 s, 56 °C for 30 s, 72 °C for 30 s, and a ramp rate of 1.6 °C/s, followed by a melting curve analysis. Predesigned LNA primer pairs were purchased from Qiagen for each of the selected miRNAs (mmu-miR-802-5p, mmu-miR-194-5p, mmu-miR-92a-1-5p, mmu-miR-205-5p, mmu-miR-181a-1-3p) and miR-16-5p was used as an endogenous control to normalize the expression levels. RT-qPCR was performed in 394-well plates, with each sample performed in triplicate, and a no-template control was included for each amplification. Determination of the threshold cycle was performed using the QuantStudio™ Real-Time PCR software (Applied Biosystems), and the quantifications performed by the ∆∆Ct method. The results are presented as fold-change.

### 4.9. In Situ Hybridization (ISH)

ISH was used to validate the expression of miR-802-5p, miR-194-5p, miR-92a-1-5p, miR-205-5p, and miR-181-1-3p in mouse brain parenchyma, as well as in b.End5 and 4T1 cells used in the in vitro model of BCBM. ISH was performed using a 5′-3′ double digoxygenin (DIG)-labelled probe, containing locked nucleic acid (LNA) and 2′-O-methyl (2′OMe) RNA modified oligonucleotides (Qiagen, Dusseldorf, Germany).

ISH was performed as previously described [72]. Briefly, paraffin-embedded mouse brain tissue sections were deparaffinized and heat-mediated antigen retrieval was performed with sodium citrate buffer pH 6.0 (microwaved for 15 min). Then, the sections were hybridized with correspondent probes (80 nM, Qiagen, Dusseldorf, Germany) at the hybridization temperature of each miRNA for 1 h. The hybridization signal was detected with alkaline phosphatase (AP)-labelled anti-DIG (1:1500, Roche, Penzberg, Germany) for 1 h at room temperature. Nitro-blue tetrazolium chloride/5-bromo-4-chloro-3′-indolyphosphate p-toluidine salt (NBT/BCIP) (1:50, Roche) was used as a chromogenic substrate for AP.

For fixed cells, the procedure employed is equivalent to that described for brain sections, without the deparaffination and antigen retrieval steps. Fixed cells were permeabilized with 0.3% of Triton-x for 15 min on ice and hybridized with correspondent probes (50 nM) at the hybridization temperature of each miRNA for 1 h. The hybridization signal was detected by adding AP-labelled anti-DIG (1:1500) for 1 h at room temperature. NBT/BCIP (1:50) was used as a chromogenic substrate for AP. Nuclei counterstaining was performed with Hoechst 33,342 dye (1:1000, Thermo Fisher Scientific, Waltham, MA, USA), for 10 min at room temperature.

Negative control assays were performed without probes for both tissues and cells.

Images were acquired at the Faculty of Sciences, University of Lisbon, Microscopy Facility, using a bright field microscope (Olympus, model BX51) with an integrated digital camera (Olympus, model DP50) with mercury fluorescence illuminator, and Nomarski/DIC Prism for Transmitted Light. Ten fields of the cranial hippocampus of each animal (*n* = 3) and ten fields of each cell culture condition (*n* = 3) were processed and analyzed using ImageJ 1.29x software (National Institutes of Health, Bethesda, MD, USA).

### 4.10. Statistical Analysis

Results were analyzed using GraphPad Prism^®^ 6.0 (GraphPad Software, San Diego, CA, USA) and are expressed as mean ± SEM. The results represent the average of three independent experiments (*n* = 3). Two-tailed Student’s *t*-test was performed when only two conditions where compared (e.g., one independent variable) or one-way ANOVA (for multiple comparisons with data with a normal distribution) was performed for comparisons between cell culture conditions and time points. *p*-values less than 0.05 were considered statistically significant.

## 5. Conclusions

The present studies revealed changes in the content of EVs and miRNAs in the peripheral circulation during BCBM development, with distinctive features of miRNAs in early and EVs in late stages of the metastatic process. In fact, an increased content of TfR-positive EVs at later stages of BCBM development was observed, indicating that BBB-derived EVs may constitute a new biomarker of advanced stages of the disease. On the other hand, the differential expression of miR-802-5p, miR-194-5p, miR-92a-1-5p, miR-205-5p and miR-181a-1-3p in plasma samples, paralleled by corresponding brain expression alterations in early stages of the disease, point to these miRNAs as candidates for precocious and/or predictive biomarkers of BCBM formation. Despite possible additional contributions of different brain cell types, BMECs appeared to account for the decreased levels of miR-194-5p, whereas BCCs were shown to significantly contribute to the increased content of miR-205-5p. Collectively, this work reveals TfR-positive EVs and specific miRNAs as novel biomarkers of BCBM in liquid biopsies, with their clinical relevance to be pursued, specifically reflecting already established metastases and incipient ones, respectively.

## Data Availability

The datasets used and/or analyzed during the current study are available from the corresponding author on reasonable request.

## Supplementary Materials

The following are available online at https://www.mdpi.com/article/10.3390/ijms22105214/s1.
