# Supplementary material for: MicroRNAs and Extracellular Vesicles as Distinctive Biomarkers of Precocious and Advanced Stages of Breast Cancer Brain Metastases Development
Source: Int J Mol Sci. 2021 May 14;22(10):5214. doi: 10.3390/ijms22105214 (PMC8155987; doi:10.3390/ijms22105214)
Supplement: Supplementary file 1 [file ijms-22-05214-s001.zip › ijms-1212293-supplementary.pdf]

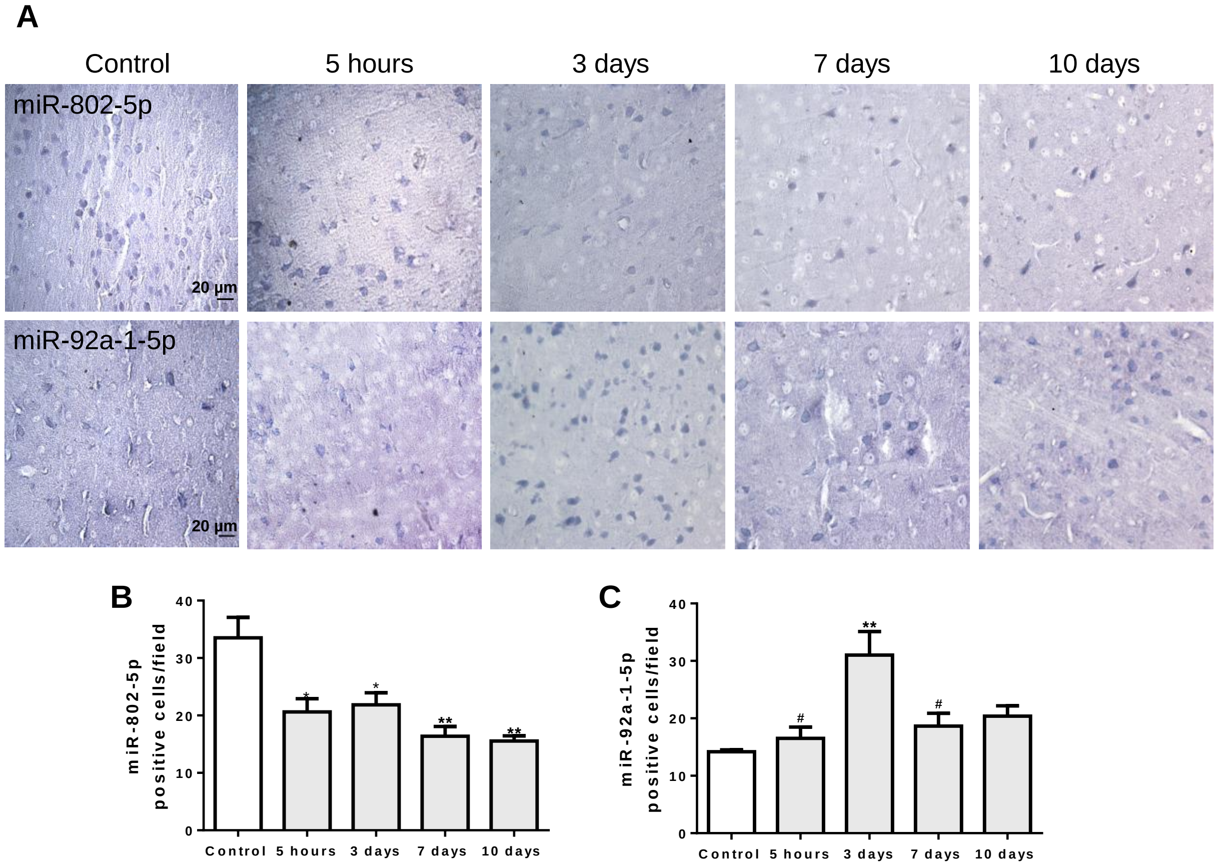

Figure S1. miR-802-5p and miR-92a-1-5p expression varies along breast cancer brain metastasis formation. 4T1 cells or vehicle (Control) were inoculated in the carotid artery of female Balb/c mice and after 5 hours, 3, 7 or 10 days brains harvested upon sacrifice. Sections of cranial hippocampus were processed for in situ hybridization (ISH) analysis of one of the predicted downregulated (miR-802-5p) and upregulated (miR-92a-1-5p) miRNAs, evidenced by a blueish coloration expression of these miRNAs in brain cells (**A**). Semi-quantititative analysis of the number of each miRNA positive cells per field highlighted miR-802-5p downregulation for all the timepoints studied comparatively to control (**B**) while miR-92a-1-5p upregulation was only significant after 3 days (**C**). Scale bar: 20 µm. Statistical differences are denoted as *p<0.05, **p<0.01, vs. control or as #p<0.05 vs. 3 days by one-way ANOVA. Data represented are means ± SEM, n=3.
